# Oncolytic activity of naturally attenuated herpes-simplex virus HF10 against an immunocompetent model of oral carcinoma

**DOI:** 10.1016/j.omto.2020.12.007

**Published:** 2020-12-19

**Authors:** Gaku Takano, Shinichi Esaki, Fumi Goshima, Atsushi Enomoto, Yoshimi Hatano, Haruka Ozaki, Takahiro Watanabe, Yoshitaka Sato, Daisuke Kawakita, Shingo Murakami, Takayuki Murata, Yukihiro Nishiyama, Shinichi Iwasaki, Hiroshi Kimura

**Affiliations:** 1 Department of Otolaryngology, Head and Neck Surgery, Nagoya City University Graduate School of Medical Sciences and Medical School, Nagoya, Japan; 2 Department of Virology, Nagoya University Graduate School of Medicine, Nagoya, Japan; 3 Department of Pathology, Nagoya University Graduate School of Medicine, Nagoya, Japan; 4 Department of Virology and Parasitology, Fujita Health University School of Medicine, Aichi, Japan

**Keywords:** oncolytic viotherapy, herpes simplex virus, HF10, tongue cancer, murine oral carcinoma, 4-nitroquinoline 1-oxide

## Abstract

Prognosis for advanced oral carcinoma remains poor. Oncolytic virotherapy uses replication-competent viruses to infect and kill only the tumor cells. However, it has been difficult to investigate the oncolytic activity of viruses against oral carcinomas in mouse models. This study established a mouse model of oral cancer and investigated the *in vitro* and *in vivo* anti-tumor effects of HF10, a highly attenuated, replication-competent herpes simplex virus (HSV)-1. Mouse tongue cancer was induced by injecting 4-nitroquinoline 1-oxide into the mouse tongue. The murine oral cancer cell line isolated from this tumor, named NMOC1, formed invasive carcinoma within a week when injected into mouse tongue. HF10 successfully infected, replicated, and spread in the cancer cells *in vitro*. HF10 was able to kill cancer cells isolated from human or mouse tongue tumor. HF10 injection into tongue carcinomas prolonged mouse survival without any side effects or weight loss. Intertumoral injection of GFP-expressing HF10 confirmed that viral spread was confined within the tumors. Immunohistochemical staining showed that HF10 induced infiltration of CD8-positive T cells around HSV-infected cells in the tumor mass, implying increased anti-tumor immunity. We successfully established an oral cancer cell line and showed that HF10 is a promising therapeutic agent for oral cancer.

## Graphical Abstract

**Figure:**
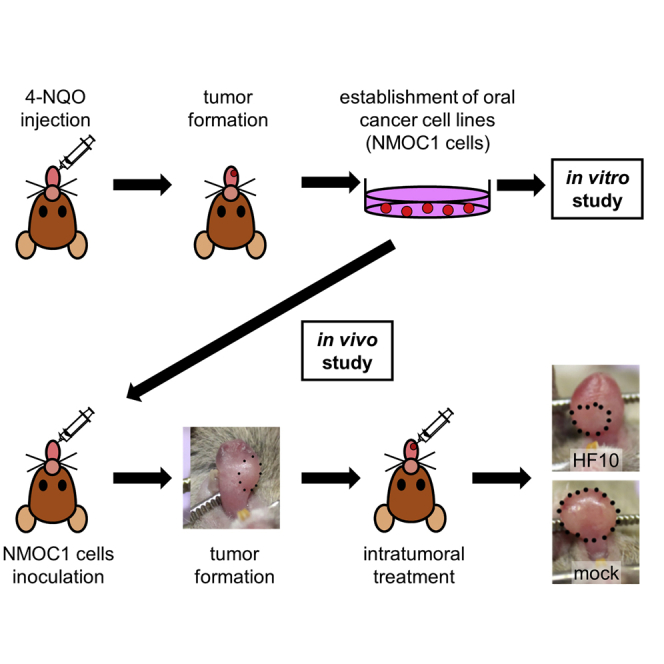

## Teaser

Immune responses can enhance anti-tumor capacity of viruses; therefore, full investigation of oncolytic virotherapy requires use of non-immunodeficient mice. Since no commercial cell lines are available, we established a murine model for oral carcinoma and evaluated oncolytic activity of HF10, naturally attenuated herpes simplex virus, *in vitro* and *in vivo*.

## Introduction

The use of viruses for cancer treatment has been reported as early as the 1950s.^1^ In 1974, a study in Japan that used the mumps virus for treating terminal cancer had positive results in 37 out of 90 patients;^2^ however, further clinical studies were not performed. After a genetically attenuated virus displayed effectivity in treating a mouse model of glioma in 1991,^3^ more researchers focused on using attenuated viruses for cancer treatment in what is now called “oncolytic virotherapy.” In 2015, Talimogene laherparepvec (T-VEC) was approved by the U.S. Food and Drug Administration as the first oncolytic virus for the treatment of advanced inoperable malignant melanoma.^4^ HF10 (also called canerpaturev) is a highly attenuated, replication-competent mutant of the herpes simplex virus (HSV)-1 that was isolated in our laboratory. We have previously shown the anti-tumor effects of HF10 in various tumor models.^5^, ^6^, ^7^, ^8^, ^9^ Recently, several clinical trials have demonstrated the safety and efficacy of HF10.^10^

Oral cancer is among the ten most common cancers in the world, with over 350,000 diagnosed cases and 175,000 deaths each year.^11^ The major risk factors for oral cancer are smoking and alcohol.^12^ Although treatment has advanced through the combination of surgery, radiotherapy, and chemotherapy, prognosis remains poor; the overall 5-year survival rates for cancers of the tongue, oral cavity, and oropharynx are around 50%–60%.^13^ Oral cancer arises in the oral mucosa and metastasizes to cervical lymph nodes; since these sites are accessible for injection, local injection of oncolytic viruses is potentially a novel therapeutic strategy. Some oncolytic viruses for oral cancer have already been reported.^14^^,^^15^ Since oncolytic activity can be affected by the tumor itself and by the host immune system, *in vivo* studies are essential for the complete investigation of anti-tumor effects. In most studies, human oral squamous cell carcinoma (OSCC) cell lines were injected subcutaneously in nude mice.^16^ However, anti-tumor immune responses cannot be investigated in this mouse model, since those mice lack T cells. To assess the immune responses as well as the oncolytic activity of a virotherapy candidate, a mouse model of OSCC with a normal immune system is necessary; however, no commercial cell lines for the establishment of murine oral cancer *in vivo* are available.^17^, ^18^, ^19^

Therefore, in this study, we first aimed to establish an oral carcinoma mouse model. Subsequently, we used this model to investigate the anti-tumor effects of HF10 against OSCC *in vitro* and *in vivo*. The findings we report here will be of use for further studies and for the potential treatment of oral carcinoma.

## Results

### Establishment of the oral tumor model

**Figure 1:**
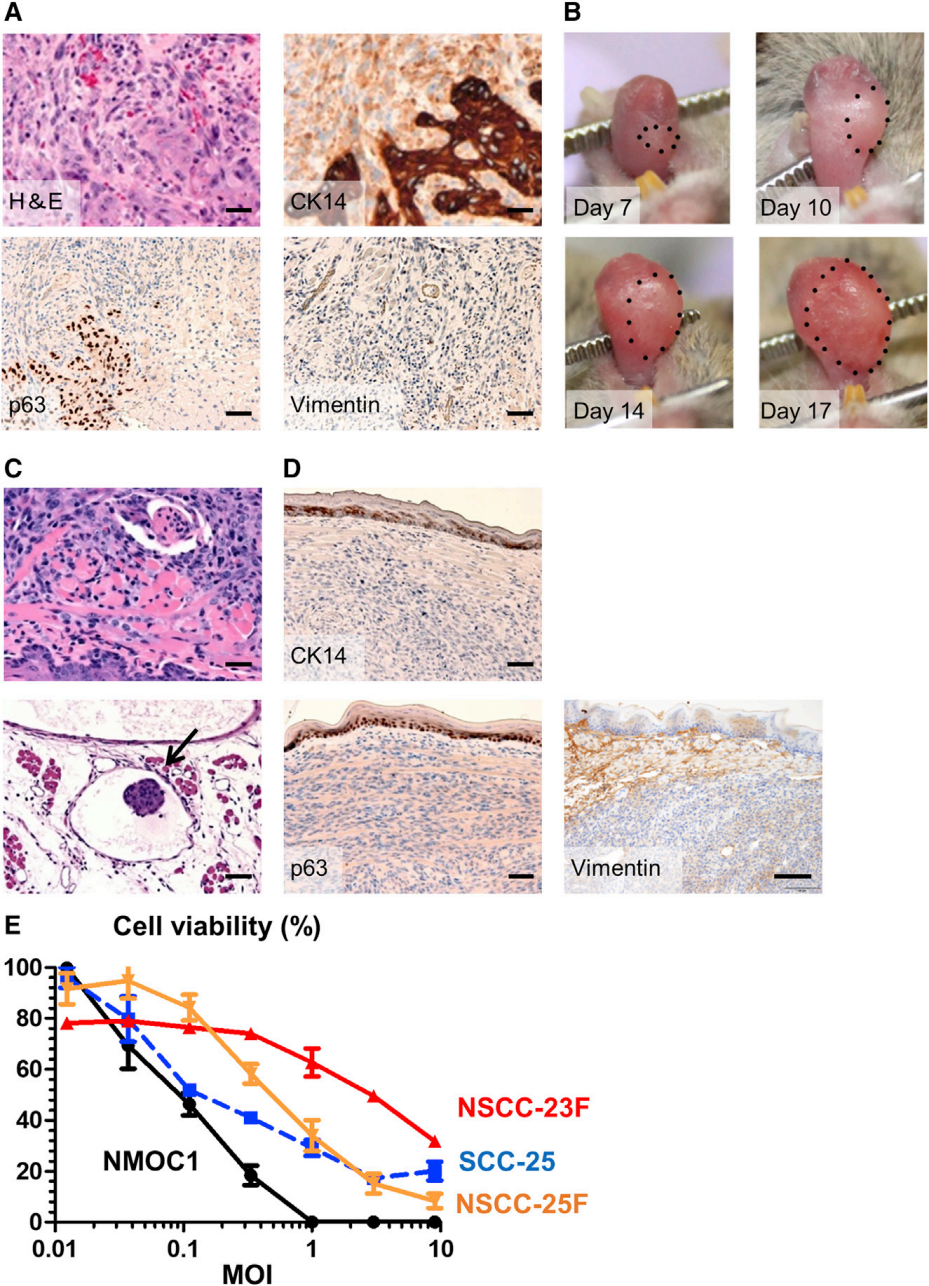
Establishment of the oral tumor model (A) Histopathological analysis of 4-NQO-induced oral tumor (primary tumor). H&E staining shows invasive squamous cell carcinoma of the tongue. Immunohistochemical staining shows that oral tumors were positive for p63 and CK14 and negative for vimentin. (B) Representative pictures showing oral tumor. Steady growth of the tongue tumor was observed over time. (C and D) Histopathological analysis of oral tumor on day 14. With H&E staining, massive tumors that deeply invaded into the skeletal muscle fibers of the tongue were observed. Arrows show metastasis to lymphatic vessels. Immunohistochemical staining showed that tongue tumors were negative for p63, CK14, and vimentin. Scale bars, 50 μm. (E) HF10 cytotoxicity in oral cancer cells *in vitro*. The viability of HF10-infected cells was analyzed 48 h post-infection. Viability of the HF10-treated cells is expressed as percentage relative to that of the uninfected control. Cytotoxicity increased in an MOI-dependent manner. Data are presented as the mean ± SEM.

Previous murine models of oral cancer were generated by the addition of the carcinogen 4-nitroquinoline 1-oxide (4-NQO) to drinking water for 6 months.^20^ However, the tumors grew at different times and formation sites. Therefore, in this study, we aimed to establish a stable model of murine oral cancer. Injection of 4-NQO directly into the mouse tongue induced tongue tumor formation in 4 weeks (Figure 1A). Pathological observation revealed that the tumor was invasive squamous cell carcinoma. Immunohistochemical staining revealed the tumor cells were positive for cytokeratin 14 (CK14) and p63 but negative for vimentin, all of which confirmed the tumor had similar characteristics to human OSCC.

Next, tumorigenicity of NMOC1 cells, a cancer cell line obtained from cultures of tongue tumor, were inoculated into the tongue to assess their *in vivo* tumorigenicity. Tumors formed in the tongues of all mice within 7 days, and daily tumor growth was observed (Figure 1B). To examine the pathological features of the tumor, the tongue tumors were removed 14 days after inoculation of NMOC1 cells. Pathological examination showed that massive tumors formed in the tongue and deeply invaded into the tongue skeletal muscle fibers (Figure 1C). The tumor also metastasized to lymphatic vessels. Immunohistochemical staining showed that the tumor was negative for vimentin and lost the expression of CK14 and p63 (Figure 1D), which suggested dedifferentiation of tongue tumors post-*in vivo* passage.

### Cytotoxicity of HF10 *in vitro*

Next, we examined the cytotoxicity of HF10 *in vitro*. NMOC1 cells were infected with HF10 at a variety of multiplicity of infections (MOIs). At 48 h post-infection, 80% of the cells were killed by an MOI of 0.3 (Figure 1E). Then, we examined the cytotoxicity of HF10 in other human oral cancer cells. HF10 cytotoxicity increased with increasing MOIs in SCC-25, NSCC-23F, and NSCC-25F cells, indicating oncolytic activity of HF10 for human and mouse OSCCs *in vitro*.

### Expression of HSV entry receptors into OSCC cells

Nectin-1 and herpesvirus entry mediator (HVEM) are two major cellular receptors that mediate HSV-1entry. All OSCCs derived from tongue tumors expressed nectin-1 and HVEM (Figure S1); expression of nectin-1 and HVEM in NSCC-25F cells and that of HVEM in NMOC1 cells was stronger.

### Replication ability of HF10 in NMOC1 cells

**Figure 2:**
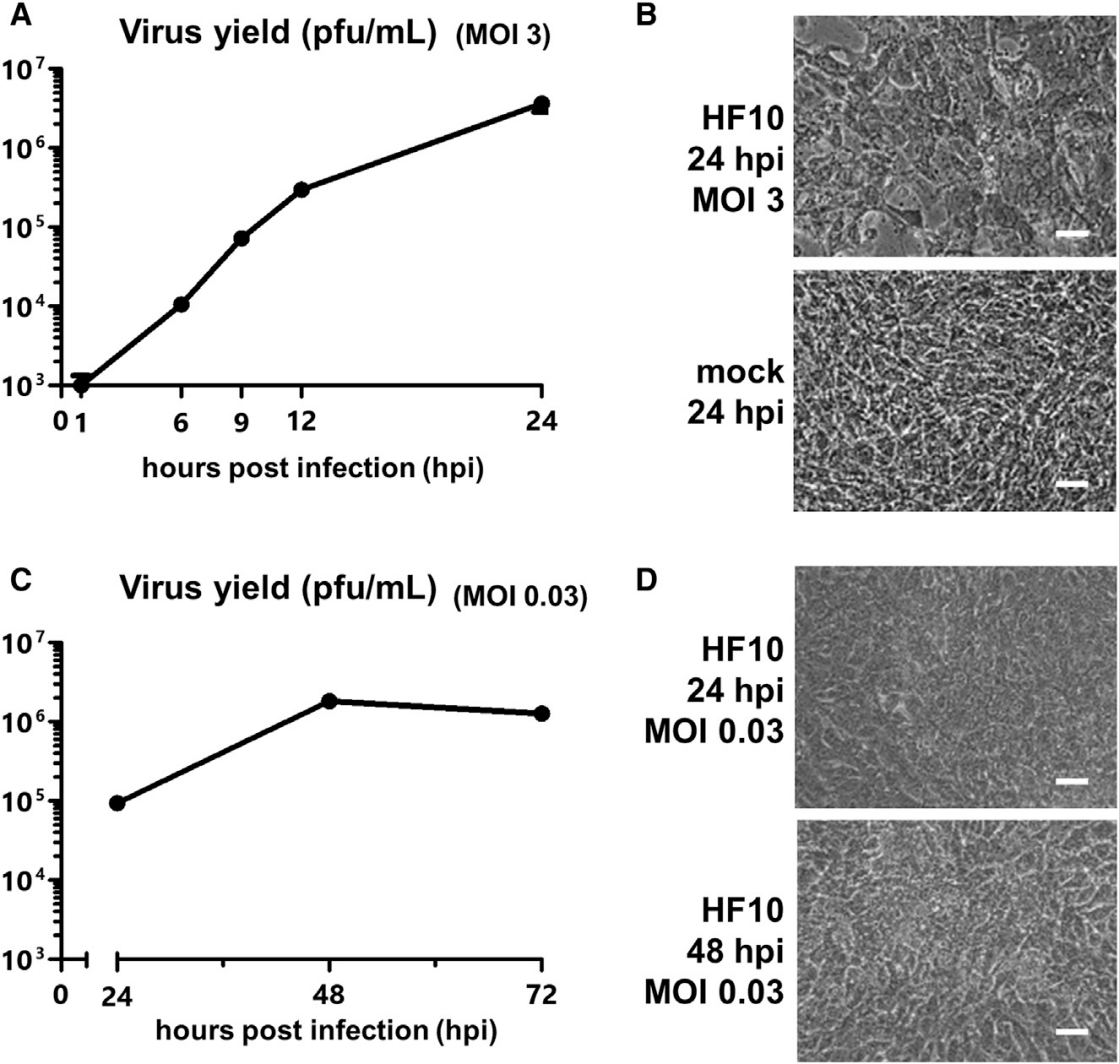
Spread and replication efficiency of HF10 in NMOC1 cells (A) Single-step growth of HF10 (MOI of 3) in NMOC1 cells. (B) Representative images of NMOC1 cells infected with HF10 or mock (PBS) at an MOI of 3 at 24 h post-infection. CPE was observed after infection. (C) Multi-step growth of HF10 (MOI of 0.03) in NMOC1 cells. (D) Representative images of NMOC1 cells infected with HF10 at an MOI of 0.03 at 24 and 48 h post-infection. CPE was observed following infection. Scale bars, 25 μm.

We next assayed the replication ability of HF10 in NMOC1 cells. Infection with HF10 at an MOI of 3 showed that HF10 could replicate well in NMOC1 cells (Figure 2A) and could induce cytopathic effects (CPEs) in NMOC1 cells (Figure 2B). HF10 could also replicate well at an MOI of 0.03 (Figure 2C) and could induce CPEs (Figure 2D). NMOC1 cells were infected with HF10-GFP at an MOI of 0.03 to visualize virus replication. The number of GFP-positive cells increased up to 48 h post-infection (Figure S2), which coincides with the results of the replication assay of HF10.

### Therapeutic efficacy of HF10 in the oral tumor model

**Figure 3:**
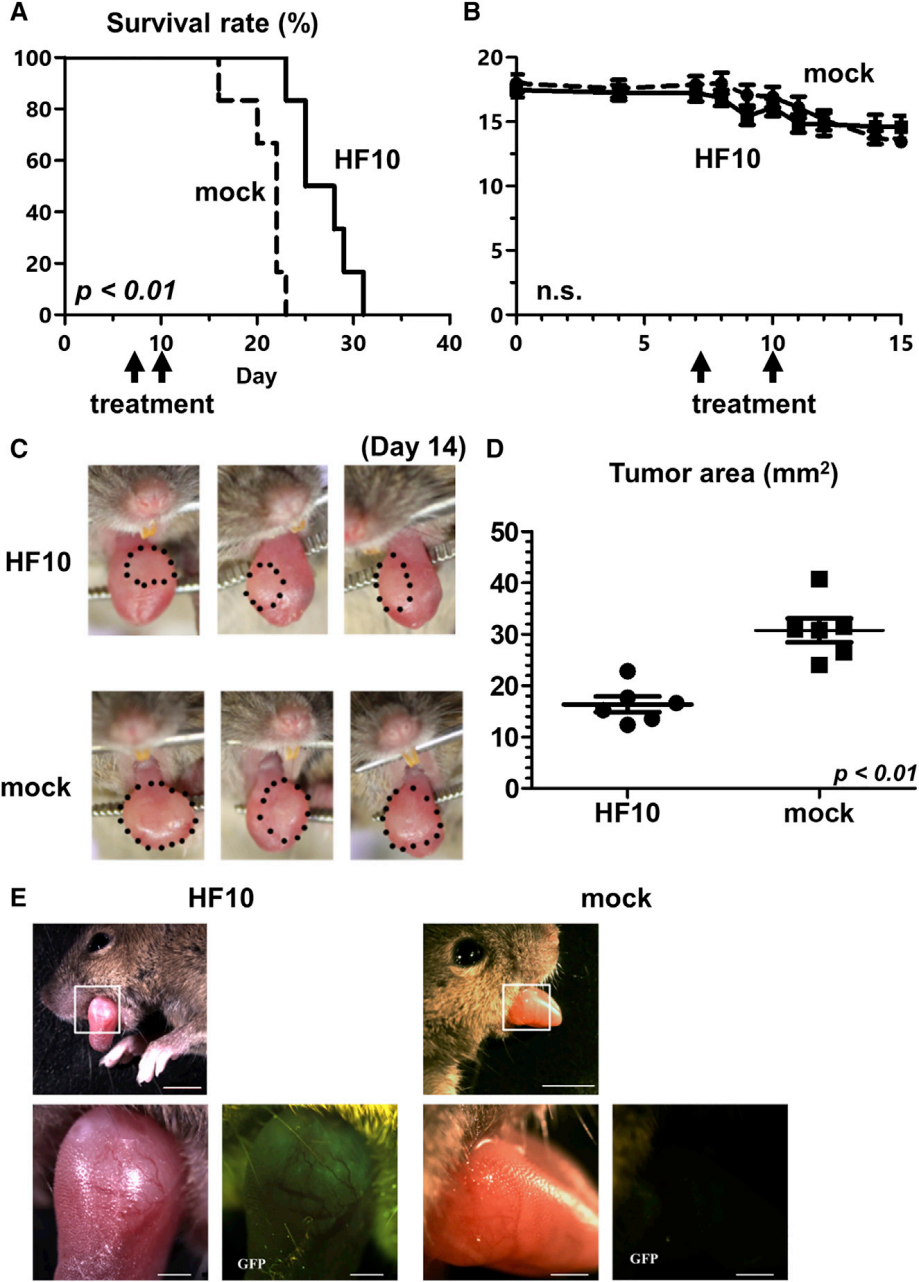
Therapeutic efficacy of HF10 in the *in vivo* oral tumor model (A) Survival analysis shows that HF10 treatment significantly prolonged survival compared to mock (PBS) treatment (n = 6, p < 0.01). (B) Daily body weight observation showed there was no difference in average body weight between the two groups. (C) Tongue tumors of 3 mice from each group on day 14. (D) The HF10 group had lower tumor volume than the mock group (n = 6, p < 0.01). (E) GFP expression in oral tumor after infection with HF10-GFP or mock (PBS) on day 21. GFP expression was detected within the oral tumor that was infected with HF10-GFP; no expression was detected in the mock group. Scale bars, 5 mm (upper images) and 1 mm (lower images). Note, a piece of food particle on the tongue of the mock mouse reflected exciting light. Data are presented as the mean ± SEM.

To investigate the oncolytic activity of HF10 *in vivo*, HF10 was injected into the NMOC1 cell-induced tongue tumor in mice. Compared to the mock treatment, HF10 injection significantly prolonged mouse survival as shown in Figure 3A (p < 0.01). No weight loss or side effect was observed in the two groups (Figure 3B). As shown in Figures 3C, 3D, and S3, tumor regression was greater in the HF10-treated group than in the mock-treated group.

### Distribution of HF10 in the oral tumor model

To visualize the distribution of HF10 in the mouse model *in vivo*, HF10-GFP was injected into the tongue tumors of mice. Twenty-four hours after HF10-GFP injection, GFP expression was observed within the oral tumor and not in other regions, while no GFP expression was observed in the mock group (Figure 3D). Seventy-two hours post-injection, GFP expression was not observed (data not shown). These results indicate that HF10 was selectively distributed within the tongue tumor.

### Induction of lymphocyte infiltration into tumors by HF10

**Figure 4:**
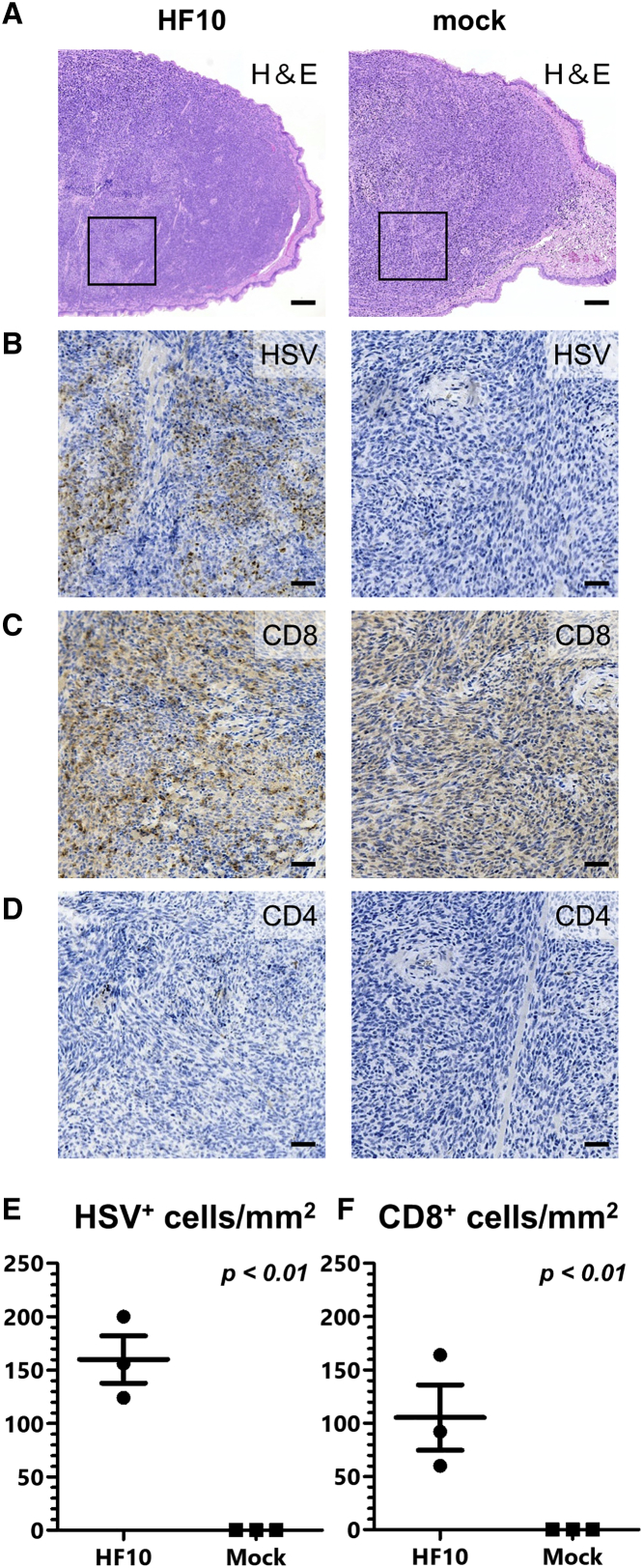
HF10-induced lymphocyte infiltration of oral cancer tumors (A) Histopathological analysis of tumors from HF10- and mock-treated (PBS) groups by H&E staining on day 14. Tumor necrosis was detected after HF10 treatment. Scale bars, 200 μm. (B–D) Immunohistochemical staining for the HSV-1 antigen, CD8, and CD4 around the necrotic region. The HSV-1 antigen was observed in the necrotic region following HF10 treatment where infiltration of CD8-positive T cells was observed. However, CD4-positive T cells were not observed. Scale bars, 50 μm. (E and F) Number of HSV-positive cells and CD8-positive cells in three mice (three area per mouse). CD8-positive cells were detected in the HSV-infected area. Data are presented as the mean ± SEM.

We next examined the immune response induced by HF10 treatment. Tongue tumors from the mice were collected and examined histopathologically. In the HF10-treated group, tumor necrosis was observed, while there were no histological changes observed in the tumors of the mock group (Figure 4A). Immunohistochemical staining for HSV antigen-stained cells detected the antigen within the tumors of the HF10-treated mouse (Figures 4B and 4E). Infiltration of CD8-positive cells was detected only in the HF10-treated group (Figures 4C and 4F), while no CD4-positive cells were detected in either group (Figure 4D).

## Discussion

Initially, oncolytic virotherapy research was focused on the tumor-shrinking properties of viruses. However, oncolytic viruses were later revealed to increase anti-tumor systemic immunity as well.^5^^,^^21^^,^^22^ Nude mice or mice with severe combined immunodeficiency (SCID) are often used to show the anti-tumor activities of agents; however, immune system involvement cannot be assessed using these mice. SCC-VII cells, derived from the murine abdominal wall, are the only cell lines available for studying squamous cell carcinoma.^23^ Therefore, most murine oral cancer *in vivo* studies use SCC-VII cells for tumor establishment,^24^^,^^25^ which may not be representative of oral carcinoma. Another murine model of oral cancer was generated by the addition of the carcinogen 4-NQO into drinking water.^20^ However, for this strategy, approximately 6 months is necessary to grow tumors, and the tumors grow at different times and formation sites, making it unsuitable for evaluating the therapeutic effects of anti-cancer agents.

Therefore, in this study, we aimed to establish a stable model of murine oral cancer. Injection of 4-NQO directly into the mouse tongue induced invasive carcinoma. Tumor formation was faster and shared similar pathology with previous reports.^19^^,^^20^ The tumors were positive for CK14 and p63 and negative for vimentin, which indicates the formation of squamous cell carcinoma. The NMOC1 cells that were isolated from the tongue tumor showed tumorigenicity when injected into mouse tongue. The NMOC1 cells formed tongue tumors after a short period of time (approximately 4 to 5 days), making our mouse model suitable for examining the therapeutic effect of drugs *in vivo*.

Among the principles of oncolytic virotherapy is the use of an oncolytic virus that infects and replicates in only cancer cells and destroys the cancer cells without harming normal cells. HF10 was isolated from an HSV-1 strain HF in our laboratory.^26^ We have demonstrated the potential of HF10 as an oncolytic virus against head and neck, colorectal, ovarian, and breast cancers as well as melanoma in various mouse models.^6^, ^7^, ^8^^,^^24^ In this study, HF10 was able to kill cancer cells isolated from either human or mouse tongue tumor. HF10 also decreased the volume of tongue tumors *in vivo* and prolonged mouse survival without causing weight loss or any side effects. HF10 successfully infected the NMOC1 cells, replicated in the cells, and spread to the surrounding cells. The spread of HF10 was also confined within the tongue tumors in the mouse model. These results are in line with the principles of oncolytic virotherapy, showing that HF10 is indeed a good candidate for cancer treatment.

Oncolytic virotherapy also affects the anti-tumor immune response.^27^ Intratumoral injection of oncolytic viruses promotes infiltration of CD8-positive T cells into the tumor, resulting in enhanced anti-tumor effects.^28^^,^^29^ Infiltration of CD8-positive T cells into tumors is associated with longer disease-free survival and overall survival of patients with head and neck cancers.^30^ In this study, CD8-positive T cells were detected in tongue tumors following HF10 treatment, while CD4-positive T cells were not, both of which were in accordance with our previous studies.^5^^,^^24^ We could not conduct a flowcytometric analysis since the tongue tumors were too small to remove from normal tongues. Instead, we counted CD8-positive cells using immunohistochemistry.

First-in-human studies of HF10 have indicated viral replication and oncolytic ability against head and neck cancer after intratumoral injection.^31^ Since then, further clinical trials (phase I/II) of HF10 for patients with refractory head and neck cancer or solid cutaneous tumors have been conducted (NCT01017185) in the United States. In that trial, HF10 resulted in stable disease (SD) in 33% of patients and resulted in decreased tumor size in some patients and fewer adverse events compared with T-VEC treatment.^10^ Furthermore, in the trial, oncolytic activity of HF10 could be observed in the recurrent tumors that arose after conventional therapies. Since most oral carcinomas arise from the mucosa, the tumors are accessible for injection of oncolytic viruses. Oncolytic viruses use a different mechanism compared to other conventional therapies to destroy cancer cells;^32^ therefore, oncolytic virotherapy is a potent anti-cancer therapy. To maximize the benefits of oncolytic virotherapy for oral carcinoma, more research must be undertaken to enhance the anti-tumor activities of oncolytic viruses.

In conclusion, we have successfully established a new oral carcinoma cell line, NMOC1, that can be used to form tongue tumors in a mouse model. This is one of the first murine oral carcinoma models with a normal immune system. We have also demonstrated the oncolytic activity of HF10 in tongue tumor models. HF10 replicated in the tumor cells and killed oral carcinoma cells *in vitro* and *in vivo*. These results show that HF10 is a promising agent for treatment of oral carcinomas.

## Materials and methods

### Animals

Specific pathogen-free, 6-week-old, female C3H/HeSlc mice (Japan SLC, Hamamatsu, Japan) were used in this study. All mice were given food and water *ad libitum* and were kept under a controlled 12-h light/dark cycle at 22°C. All animal studies were approved by the University Committee in accordance with the guidelines issued by the Animal Center at Nagoya University School of Medicine.

### Viruses

HF10 is an attenuated, replication-competent mutant strain of HSV-1, and its structure has been previously described.^26^^,^^33^ In brief, the HF10 genome has a 3.9-kb deletion at the end of the unique long region and a 2.3-kb deletion. Sequence analysis showed that HF10 lacks the expression of functional UL43, UL49.5, UL55, and UL56 and the latency-associated transcript. HF10 was propagated in Vero cells (African green monkey kidney cells) and stored as aliquots at −80°C. Viral titers were determined by plaque assays with Vero cells and are expressed as plaque forming units (pfu)/mL. HF10-GFP was made by inserting green fluorescent protein (GFP) gene into HF10 under the control of the cytomegalovirus (CMV) promoter, as previously described.^34^

### Cell lines

Vero cells were obtained from Riken Cell Bank (Tsukuba, Japan). Vero cells were grown in Eagle’s minimal essential medium (MEM; Nissui Pharmaceutical, Tokyo, Japan) containing 100 U/mL penicillin, 100 mg/mL streptomycin, and 10% fetal calf serum (FCS). SCC-25, a tongue squamous cell carcinoma cell line, was obtained from the American Type Culture Collection (CRL1628; Rockville, MD, USA). SCC-25 cells were maintained in 1:1 mixture of Dulbecco’s modified Eagle’s medium (DMEM) and Ham’s F12 medium (Wako Pure Chemical Industries, Osaka, Japan) containing 1.2 g/L sodium bicarbonate, 100 U/mL penicillin, 100 mg/mL streptomycin, and 10% FCS.

NSCC-23F and NSCC-25F cells are human OSCC cells isolated from tongue squamous cell carcinomas at the Nagoya City University Hospital. To establish NSCC-23F and NSCC-25F cells, a part of the surgical specimen was removed, minced, digested with 0.1% trypsin at 37°C for 30 min, and cultured in DMEM supplemented with 10% FCS and antibiotics. Tissue collection and culture were approved by the Institutional Review Board at Nagoya City University Hospital. Cells at passage numbers less than 15 were used for experiments.

### Establishment of the mouse model for oral carcinoma

The tongues of mice (n = 3) under chloral hydrate anesthesia (360 mg/kg) were injected with 12.5 μg of 4-NQO (25 μL). 4-NQO was injected twice a week for 4 weeks. After confirmation of tumor formation, NMOC1 wells were prepared by first removing the tongue tumors after lethal anesthesia with chloral hydrate (720 mg/kg). The tumors were digested with 0.25% trypsin at 37°C for 15 min, washed with PBS, and cultured in high-glucose DMEM (Sigma-Aldrich, St. Louis, MO, USA) with 10% FCS, 100 U/mL penicillin, and 100 mg/mL streptomycin. Passage numbers less than 15 were used for experiments.

To confirm tumorigenicity, NMOC1 cells (1 × 10^6^) were injected into the tongues of mice under anesthesia using chloral hydrate. Fourteen days after inoculation, tongues were removed as described above. The tongues were fixed in 10% buffered formalin, and paraffin-embedded 4-μm serial sections were subjected to hematoxylin and eosin (H&E) staining and immunohistochemical staining.

### Histopathological and immunohistochemical analysis of the tongue tumors

To examine the 4-NQO-induced tongue tumor, it was removed after lethal anesthesia and fixed in 10% buffered formalin. Paraffin-embedded tissues were sliced into 4-μm-thick serial sections by a microslicer. H&E staining and immunohistochemical staining were performed on serial sections, as previously described.^35^^,^^36^ In brief, H&E staining was conducted using conventional Mayer’s H&E stain. For immunohistochemical staining, rabbit monoclonal anti-CK14 antibody (ab7800, Abcam, Cambridge, UK), rabbit polyclonal anti-p63 antibody (GTX102425, GeneTex, Irvine, CA, USA), and mouse monoclonal anti-vimentin antibody (TX100619, GeneTex) were used as primary antibodies, goat anti-rabbit immunoglobulin conjugated to peroxidase-labeled dextran polymer (414141F, Nichirei, Tokyo, Japan) was used as secondary antibody, and 3,3′-diaminobenzidine (DAB^+^, K3467, Dako A/S, Glostrup, Denmark) was used to visualize the complex. Specificities of the antibodies were verified using a universal negative control immunoglobulin (IS600, Dako A/S). Images of serial sections were obtained through microscopy (Olympus, Tokyo, Japan) with a CCD camera (Nikon, Tokyo, Japan).

### Cytotoxicity assay

NMOC1, SCC-25, NSCC-23F, and NSCC-25F cells were seeded into 96-well plates (5,000 cells/well), infected with HF10 at various MOIs from 0.03 to 3 the following day and incubated for 48 h. The cytotoxicity of HF10 was evaluated by MTS (3-(4,5-dimethylthiazol-2-yl)-5-(3-carboxymethoxyphenyl)-2-(4-sulfophenyl)-2H-tetrazolium) assay using CellTiter 96 AQueous one solution reagent (G3581; Promega, Fitchburg, WI, USA), according to the manufacturer’s instructions. Cytotoxicity was indicated as the reduction in cell viability with each treatment relative to the mock-infected cells. All samples were assayed in triplicate.

### Immunocytochemistry of oral OSCC cells

NSCC-23F, NSCC-25F, and NMOC1 cells were grown on coverslips, washed with PBS, fixed in 4% paraformaldehyde in PBS at room temperature for 10 min, and permeabilized in 0.1% Triton X-100 in PBS for 10 min at room temperature. For immunocytochemical staining, rabbit polyclonal anti-PVRL1/Nectin1 antibody (24713-1-AP; Proteintech Group, Rosemont, IL, USA) and rabbit polyclonal anti-TNFRSF14/HVEM antibody (ab47677; Abcam) were used as primary antibodies. Peroxidase-labeled dextran polymer (414141F; Nichirei) was used as a secondary antibody, and the complex was visualized using DAB^+^ (K3467, Dako A/S). Cell images were obtained using a microscope (Olympus) attached to a CCD camera system (Nikon).

### Spread and replication of HF10 *in vitro*

NMOC1 cells were plated on 35-mm dishes at a density of 4 × 10^6^ cells per dish in 2 mL medium. Cells were infected with HF10 at an MOI of 0.03 or 3. Supernatants were collected at the indicated time points to analyze the time course of viral production from infected cells. Viral titers were determined by plaque assays in Vero cells. All samples were measured three times. Cell images were taken with a microscope (Olympus) attached to a CCD camera system (Nikon). To visualize the spread of HF10, NMOC1 cells were plated on 35-mm dishes at a density of 4 × 10^6^ cells per dish in 2 mL of medium and infected with HF10-GFP at an MOI of 0.03. GFP-expressing cells were visualized using a Leica M205FA automated fluorescence stereomicroscope with a standard GFP filter set (Leica Mikrosysteme Vertrieb, Wetzlar, Germany) at the indicated times.

### Distribution of HF10 in the tongue tumor

NMOC1 cells (1 × 10^6^) were inoculated into the tongues of mice. Twenty days following inoculation, HF10-GFP (1.0 × 10^7^ pfu/50 μL) or sterile PBS (mock) was injected into the tongue tumor. The mice were anesthetized 24 h and 72 h post-injection, and the tongue tumors were observed using the Leica M205FA automated fluorescence stereomicroscope with the standard GFP filter set.

### Therapeutic efficacy of HF10 in the tongue tumor

NMOC1 cells (1 × 10^6^) were inoculated into the tongue (n = 12), and the mice were divided into two groups (HF10 and mock) in a random manner. HF10 (1.0 × 10^7^ pfu/50 μL) or PBS (mock) was injected twice into the tongue tumors on days 7 and 10. The general condition of the mice was monitored daily. On day 14, tongue tumors were measured and the area was calculated using the formula: (π × short axis × long axis)/4.

### Histopathological and immunohistochemical analysis following HF10 treatment

NMOC1 cells (1 × 10^6^) were inoculated into the tongue, and HF10 (1.0 × 10^7^ pfu/50 μL) or PBS (mock) was inoculated twice into the tongue tumor after 10 and 13 days. On day 14, mice were anesthetized, and the tongue tumors were collected and fixed in 10% buffered formalin. Paraffin-embedded tissues were sliced, and serial sections were subjected to H&E staining and immunohistochemical staining. Rabbit polyclonal anti-HSV antibody (ab226858; Abcam), rabbit polyclonal anti-CD4 antibody (bs-0647R, Bioss Antibodies, Woburn, MA, USA), and rabbit polyclonal anti-CD8 antibody (bs-4790R; Bioss Antibodies) were used as primary antibodies. The proceeding steps (secondary antibody and imaging) were performed as previously described. HSV-positive cells in a mm^3^ were counted and averaged from 3 slides per mouse (n = 3) using ImageJ software (v1.52p). CD8-positive cells in the same area were also counted and averaged.

### Statistical analyses

Statistical analyses were performed by using the JMP software package (v13; SAS, Cary, NC, USA). Survival data were analyzed using the Kaplan-Meier method and the log-rank test. Differences were considered statistically significant when p <0.05.

## Supplemental information

Document S1. Figures S1–S3

Document S2. Article plus supplemental information
