# Supplementary material for: Oncolytic activity of naturally attenuated herpes-simplex virus HF10 against an immunocompetent model of oral carcinoma
Source: Mol Ther Oncolytics. 2020 Dec 19;20:220–7. doi: 10.1016/j.omto.2020.12.007 (PMC7889449; doi:10.1016/j.omto.2020.12.007)
Supplement: Document S1. Figures S1–S3 [file mmc1.pdf]

OMTO, Volume *20*

Supplemental Information

Oncolytic activity of naturally attenuated herpes-simplex virus HF10 against an immunocompetent model of oral carcinoma

Gaku Takano, Shinichi Esaki, Fumi Goshima, Atsushi Enomoto, Yoshimi Hatano, Haruka Ozaki, Takahiro Watanabe, Yoshitaka Sato, Daisuke Kawakita, Shingo Murakami, Takayuki Murata, Yukihiro Nishiyama, Shinichi Iwasaki, and Hiroshi Kimura

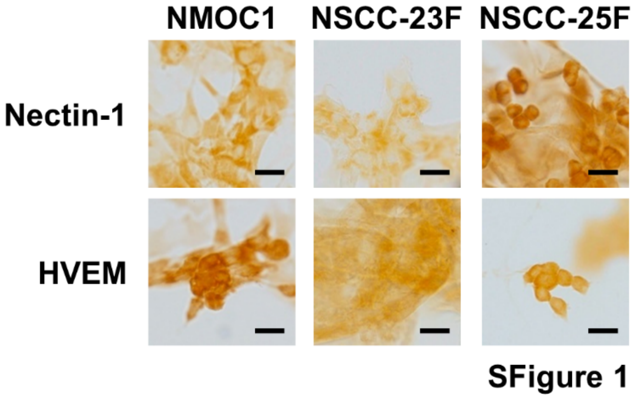

Supplementary Figure 1. Immunocytochemical staining of nectin-1 and HVEM in OSCC cells. All OSCCs expressed nectin-1 and HVEM. Expression of nectin-1 and HVEM in NSCC-25F cells and that of HVEM in NMOC1 cells was stronger.

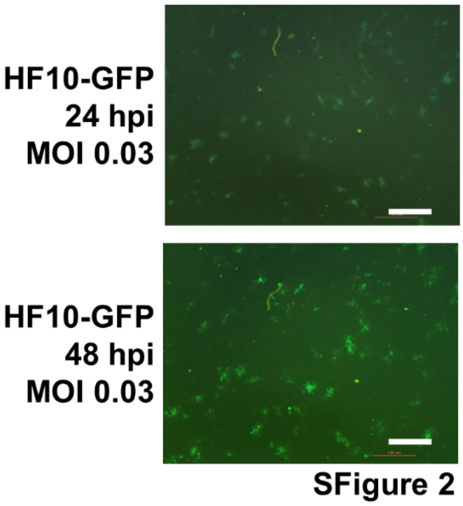

Supplementary Figure 2. Representative images of NMOC1 cells infected with HF10-GFP at an MOI of 0.03. GFP-positive cells were increased up to 48 hours post-infection. Scale bars, 500 μm.

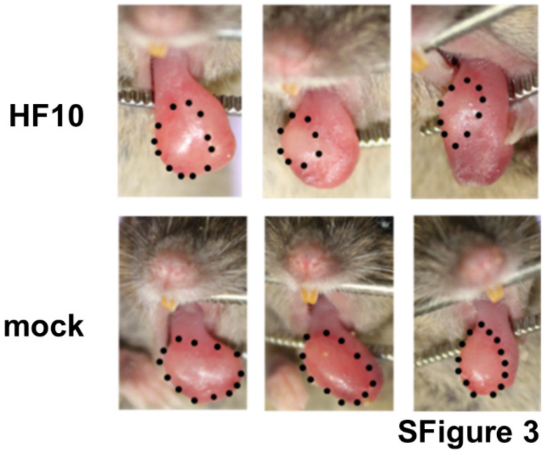

Supplementary Figure 3. Tongue tumors of the remaining 3 mice from each group on day 14.
